# Nurses’ Experience of Caring for Patients with Delirium: Systematic Review and Qualitative Evidence Synthesis

**DOI:** 10.3390/nursrep11010016

**Published:** 2021-03-05

**Authors:** Nissy Thomas, Mardhie Coleman, Daniel Terry

**Affiliations:** School of Health, Federation University Australia, Ballarat 3350, Australia; me.coleman@federation.edu.au (M.C.); d.terry@federation.edu.au (D.T.)

**Keywords:** anxiety, assessment, delirium, education, nurse, patient, peers, stress, support

## Abstract

Delirium is an acute deterioration in attention, conscious state, perception, and cognition of a person. While nurses possess the theoretical understanding of the condition, they lack insight into its early recognition and management. This systematic review aims to understand what factors influence nurses as they care for patients with delirium, and to identify best practices to improve overall clinical care. The Qualitative Evidence Synthesis (QES), as a strategy process to identify gaps in research, formulate new models or strategies for care, underpinned the review. In addition to specific inclusion and exclusion criteria, a methodological assessment, data were analysed using QES, as informed by the Joanna Briggs Institute Review process. Ten studies were identified and synthesised to generate four key themes. The themes included (1) nurse’s knowledge deficit; (2) increased workload and stress; (3) safety concerns among nurse when caring for patients with delirium; and (4) strategies used when caring for patients with delirium. Overall, the review has highlighted the need for increased delirium education and coping strategies among nurses to effectively care for patients with delirium. This may be augmented through regular education sessions to provide nurses with the confidence and competence to care for the acutely confused person.

## 1. Introduction

Delirium remains an increasing challenge for healthcare professionals, especially nurses, who are at the coalface of the healthcare industry [1]. Delirium affects the conscious and cognitive state of a person, making behaviour uncertain, prolonging hospitalisation, increasing health costs, and resulting in adverse outcomes for patients and their families [2]. Approximately 10–18% Australians over the age of 65 experience delirium at the time of hospital admission, while a further 8% develop delirium while hospitalised, particularly those with dementia are at a higher risk [3]. The incidence of delirium is greater in certain healthcare settings or situations, with more than 30% patients experiencing delirium following hip or cardiac surgery, or when receiving intensive care [3]. Caring for patients with acute delirium poses a risk for nurses due to a patient’s unpredictable behaviour and actions [4]. There is a wealth of research regarding the various medical aspects of delirium, however, little is known concerning its nursing management. Therefore, an understanding of the experiences of nurses caring for patients with acute confusion is required to give insight to and improve clinical practice.

### 1.1. Background 

Delirium is characterised by an acute deterioration in attention, conscious state, perception and cognition of a person [5]. Causes of delirium include advanced age, dehydration, hypoxia, severe illness, co-morbidities, infection, surgical procedures, medication, and metabolic abnormalities [6]. The morbidity of patient’s with delirium varies according to the patient characteristics, history of previous cognitive impairments, health care settings, and sensitivity of detection approaches such as the Confusion Assessment Methods (CAM) [7]. LaFever et al. [8], highlighted in the United States, delirium costs more than $182 million to healthcare with an associated hospital mortality rate up to 33%. The lack of appropriate nursing management of delirium can result in patient’s functional decline, greater risk of falls, and increased mortality and morbidity [3,9].

Delirium is treated as a medical emergency as it can progress rapidly, however, recognising and managing delirium can be problematic [10]. Several studies reveal nurses lacked insight into the early recognition and management of delirium with the achievement of appropriate outcomes remaining elusive [11,12,13]. Christensen [14], suggests that although nurses possess an in-depth theoretical knowledge of delirium, this knowledge is not always sufficient to detect, manage and prevent delirium. For example, among nurses, the physical aspect of caring for a patient with delirium is often the focus, while the assessment of a patient’s cognitive function is overlooked or not well implemented [6]. Despite this finding, a thorough assessment is no less important than the associated care, as it contributes significantly toward effective nursing management of patients with delirium [14]. However, there are limited studies on the assessment of risks, barriers, resources, and coping mechanisms nurses possess and use in managing a person with delirium.

Nurses provide the frontline care for patients and need to take on a more active role in the prevention, early identification, and treatment of delirium [15]. Measures, such as providing individualised care, preventing harm, managing medical issues, analysing the cause, and modifying the environment promote effective patient care, while reducing hospital costs [16,17]. However, nurses often experience stress and anxiety when assigned to patients with delirium and there is often a lack of resources to support these nurses who care for patients experiencing delirium [12,18,19,20]. This paper seeks to understand the experiences, challenges, the support required, and the need for further education to support nurses when caring for those with delirium. The results of this review seek to identify gaps, inform clinical practice, contribute to further research, and provide nurses with additional knowledge regarding the nursing care of delirium.

### 1.2. Aim 

The aim of this review is to understand what factors influence nurses as they care for patients with delirium, and to identify best practices to improve clinical practice for those with delirium.

## 2. Materials and Methods

Qualitative evidence synthesis (QES) is the primary strategy that underpins this review. Tong [21], advocates that QES enables to recognise any gaps in research, formulate new models of care, and to develop new strategies to implement care. The process of QES involves the integrative synthesis, where the data are aggregated, or summarised using common themes and remains the most appropriate method to facilitate the review [22]. As such, the search strategy aimed to obtain all relevant published studies regarding the experience of nurses caring for patients with delirium. 

### 2.1. Inclusion and Exclusion Criteria 

The reviewed studies included original qualitative studies focusing on the experience of nurses caring for patients with delirium. The inclusion criteria encompassed studies that comprised of qualified nurses (Registered and Enrolled nurses) only, while the years of experience of the nurse or the area they worked in, were not specifically considered. The review included all heath care settings such as acute care (medical and surgical wards, palliative care, oncology, and intensive care) and residential or aged care, and aged care psychiatry. Studies examining the experience of nurses caring for various types of delirium (postoperative delirium and terminal delirium) were included. Full-text studies published in English in the last 10 years were only considered. Quantitative studies and articles focusing on clinical updates were excluded, as this is a qualitative evidence synthesis, based on primary qualitative research papers and the experiences of caring for those with delirium. Studies focusing on the experience of ‘healthcare workers’ were excluded as they were generic and did not specify the type of health professionals which were included in the team.

### 2.2. Search Strategy

A three-step search strategy was undertaken for this review as outlined by the Joanna Briggs Institute reviewer manual [23]. The initial step was the identification of keywords and a general search using these words. The keywords used included “experience” OR “challenges” OR “work experience” OR “perceptions” AND “delirium” OR “acute confusion” AND “nurses” OR “registered nurses”. The second step involved a search in Cumulative Index of Nursing and Allied Health Literature (CINAHL), PubMed, Scopus and Medline using the keywords in line with the inclusion and exclusion criteria. The third and final step encompassed searching the reference list of the identified articles for additional studies, including unpublished studies.

### 2.3. Study Screening 

The articles retrieved from the literature search were screened by two reviewers (N.T and M.C.), after duplicates were removed. Both reviewers independently screened all studies based on titles, keywords, and abstracts to exclude irrelevant articles. A second round of reviews were conducted where full text articles were assessed independently and judged against the inclusion and exclusion criteria by two reviewers (N.T. and M.C.). Each study was classified as ‘include’, ‘exclude’ or ‘not sure’ in the review. Any discrepancies between the two reviewers were resolved by discussion with a third reviewer (D.T.) until consensus was achieved. 

### 2.4. Assessment of Methodological Quality 

Once screened, each qualitative paper identified was further assessed by two independent reviewers (N.T. and M.C.) for methodological quality prior to final inclusion in the review. The methodological quality process was achieved, using the standardised critical appraisal instruments from the Joanna Briggs Institute Qualitative Assessment and Review Instrument (JBI-QARI). The inclusion of a study was based on meeting, where applicable, each of the ten criteria items of the JBI-QARI [24]. Any disagreements that may have arisen between the reviewers were aimed to be resolved through discussion with a third reviewer (D.T.), however, no disagreements occurred. 

### 2.5. Data Extraction and Synthesis

Once methodological validity was completed, data were extracted to enable a detailed examination of the method, methodology, intervention, setting, geographical and cultural aspects, participants, data analysis and findings of the studies as guided by the JBI-QARI data extraction tool [24]. Data were then synthesised to generate a set of statements that represented the aggregation (Level 1 findings). These findings were then repeatedly examined and categorised depending on similarity and quality (Level 2 findings). Once grouped, the findings were further subjected to qualitative evidence synthesis where a single comprehensive set of synthesised findings was produced (Level 3 findings) as guided by the Joanna Briggs Institute review process [24].

As such, findings, such as direct participant quotes or observations, were first aggregated [24]. Then, a rating of credibility of the findings was assigned to reflect the reviewer’s perception of the degree of support each of the findings. The three levels of credibility defined by the Joanna Briggs Institute [24], include: Unequivocal (U), Credible (C) and Unsupported (NS). After the findings were rated for credibility, they were clustered according to their shared meaning. The clusters were then subjected to a qualitative evidence synthesis, whereby similar ideas within the clusters were combined and statements formulated to explain their meaning. The statement of meaning, developed from the cluster of ideas, generated the themes for synthesis, leading to a level of credibility which supported the findings.

## 3. Results

**Figure 1:**
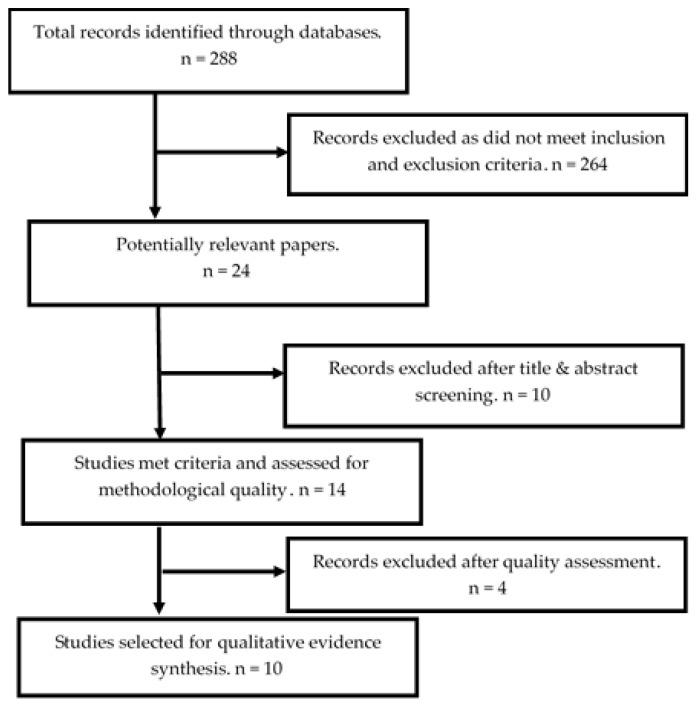
Systematic review flow chart.

The outcome of the initial search strategy yielded 24 articles for review, and after duplicates were removed and the full article of each study was screened, ten articles were excluded. The remaining 14 studies were examined for their methodological quality, which led to a further four being identified as methodologically weak, and thus excluded, as they were unable to meet, where applicable, all ten JBI-QARI criteria items. Overall, ten studies were identified and used as a basis for data synthesis and analysis (Figure 1).

Among the studies identified, it was noted they were undertaken in Australia (*n* = 3), Canada (*n* = 2), China (*n* = 1), Denmark (*n* = 1), United Kingdom (*n* = 2), and United States (*n* = 1), with the majority being conducted in acute health care settings [12,15,16,19,20,25,26,27]. All studies were qualitative in nature and their data collection was either through interview, focus group, or a combination. The only exception was Hosie et al. [18], who also used a critical incident technique along with an interview. The process is where participants are asked to recall specific incidents to identify best practice and practices where gaps may exist in care. The majority of studies used thematic analysis in an effort to meet the aims of the respective projects concerning delirium, as outlined in Table 1.

Among the ten studies, 38 findings were elucidated, and grouped into 12 categories. Several themes were created, from the meanings of the clustered ideas as informed by systematic reviews process [24]. Where applicable direct quotes from the research articles are included from the selected studies to illustrate the findings. Three of the qualitative evidence synthesis themes focused on the experience of nurses and the remainder addressed the strategies implemented in the care of the acutely confused (Table 2). 

The four themes included nurse’s deficit in delirium knowledge, leading to a lack of confidence and understanding, impacting nurse’ workload and stress, safety concerns among nurse when caring for patients with delirium, and nurses achieving care of a patient with delirium through the use of various strategies. Each are discussed in detail:

### 3.1. The Deficit in Updated Knowledge, Education and Resources 

This theme comprised of three categories which impact the ability to effectively care and manage clinical situations: Concept ambiguity regarding delirium, lack of knowledge and education, and inadequacy of resources to support nursing care. Nurses stated they were unaware about the process and course of delirium, causing difficulties in understanding their patients and in reaching the patients and their reality. Lack of knowledge and education was noted to be a major impediment to meeting patient needs. The following excerpts help to illustrate this theme:

“Assessment is usually crucial, but it just knows how to assess… I don’t know what the questions would be.”

[18] (p. 823)

“Both novice and experienced nurses talked about learning to deal with patients with delirium from watching how other nurses dealt with it. They stated that they had not learned or could not remember learning much about delirium in their formal education.”

[15] (p. 331)

“A nurse reported: ‘…when we actually have a delirious patient, and nothing seems to be working. I don’t know what would be better, I guess, and that’s what makes it very frustrating because you feel very helpless.’”

[27] (p. 333)

### 3.2. Caring for Patients with Delirium, Impacts Heavily on the Nurses’ Workload 

All studies emphasised the impact of heavy workload on the care of patients with delirium and their inability to fulfil their roles successfully, satisfactorily and within the timeframe provided. The care of patients with delirium was time consuming. Due to the shortage of staff and increased workload, nurses sought support from other sources such as peer nurses and family members. Nurses reported care strategies like listening to and following the patient with delirium consumes the nurses’ time. Caring for patients with delirium generated stress, anxiety, and mental conflicts. This was demonstrated in the following passages:

“… so, whilst one person might help the nursing staff with that confused patient, that nursing staff member still has to deal with everything around that patient like medications, treatment…”

[16] (p. 892)

“Caring for delirious patients was described as not only emotionally challenging and frustrating, but also physically exhausting…”

[20] (p. 97)

“For me it is extremely distressing, because most of the time you are short staffed, and you are on your own and have eight patients, and you have two confused patients, and you are just everywhere.”

[25] (p. 330)

### 3.3. The Unpredictable Nature of Patients with Delirium, which Creates Safety Concerns 

Nurses had concern for their own safety and the safety of their patients when delirium was evident. This led them to spend more time and resources to ensure safety of all parties and concern about their own and their patient’s welfare. These two categories led to this finding which is supported by the following excerpts:

“A lot of patients are difficult to get along. When we are trying to help them stay quiet and comfortable, they may hit us…”

[19] (p. 6)

“I’m always concerned about their safety when I go in and they are confused, not directable.”

[15] (p. 330)

### 3.4. Provision of Care Achieved Using Various Strategies

The provision of care of a patient with delirium was also shown to encompass and include the support from nursing peers and the patient families. This enabled nurses to build confidence and helped in their decision-making concerning care. In addition, the use of restraints (physical and chemical) and constant observation were also identified as strategic elements of increased care, as restraints were highlighted to be commonly used to manage patients with delirium. Overall, the strategies and resources adopted by nurses to care for patients with delirium were grouped into four categories (a) constant observation; (b) restraints; (c) family member’s involvement; and (d) peer support. Nurses agreed that acutely confused patients require constant observation to ensure safety. To protect the patient, nurses tended to raise side rails on beds, but also reported this action to be hazardous if patients climbed over them [16]. Medications were used to varying degrees, haloperidol being the most common one in an effort to restrain patients [29]. Family members were indicated to play a vital role in calming the patient and contributed in ‘bringing the patient back’ to reality so that care could be provide safely and effectively [19,27]. Nurses reported that their peers, when caring for patients with delirium, supported them in an ad hoc manner, however, they also and learned from the experiences of other nurses who had cared for others with delirium [25]. Specifically, it was highlighted in one study when it was stated:

“We sat down, and we talked about the behaviours that had been happening over the last few days…”

[18] (p. 1360)

## 4. Discussion 

The results of the studies identified the participant’s lack of knowledge and education, yet their capacity to innovate was commendable [30,31]. Nevertheless, participants reported they felt they were unclear and unprepared to care for patients with delirium, which generated anxiety [15,18]. This was further highlighted within a study conducted by Godfrey et al. [11], which had revealed delirium and delirium prevention was not included in their mandatory training or in-service education programs. Although clinical detection of delirium can be challenging, a sound understanding of cognitive assessments and the process and course of delirium will enable nurses to manage delirium effectively [22,25,32]. Furthermore, the reviewed studies highlight the importance of availability of clinical practice guidelines concerning delirium, along with access to protocols or integrated systems that translated the delirium knowledge into workplace practices [20,28]. To combat the delay in early intervention for delirium, Docherty et al., [2] (p. 12) developed a simple, yet systematic formula: “Delirium: Suspect it, spot it and stop it”.

Increased workload was another challenge faced by the participants, producing a sense of incompetence, thereby affecting their quality of care, and was seen as time consuming. This review reveals the stress and frustration encountered by nurses in trying to manage the patient and the situation [18,19,20,26]. In addition, the review revealed how the increased workload affected the productivity of the nurses and their job satisfaction [11,19,20]. Although not all facilities were understaffed, the acuity of patients with delirium increased the workload [19,25]. Furthermore, this review highlights the need for additional staff, resources, or alternative approaches to workload allocation [12,19]. 

Nurse participants reported maintaining the safety of the patient and themselves remained difficult with the aberrant behaviour of the patient with delirium led to questions about safety, which was evident in four of the studies [16,19,20,27]. Participants acknowledged being ‘victims’ of physical aggression, but with experience and clinical managerial support they had developed strategies to protect themselves [33]. Staff resources like one-on-one nursing allocation, sometimes termed ‘nurse special’, proved to be effective to manage patients with delirium [27,29]. Family members or carers supported nurses by staying with patients, calming, and reorienting them, when the nurses were unavailable or unable to refocus patients [15,19,27]. The support from family members also reduced stress, anxiety, and workload among nurses, by providing context of on the usual behaviour of the patient or what the patient may like or dislike when receiving care.

Most studies described the need for constant monitoring, the use of restraints, both physical and chemical, as methods and approaches to manage patients with delirium [19,28]. However, the legal implications of chemical and physical restraints were shown to differ significantly across the identified countries [12]. In contrast, all studies emphasised similarities when discussing the benefits of peer support, which was being used a common coping mechanism for each of the nurses [5,6]. Peer nurses played an important role in ‘backing’ and supporting each other in making ‘the right’ or more informed clinical decisions, which thereby lead to an improvement in their confidence and practice. 

### 4.1. Implications of Research

This systematic review reveals there are few studies focusing on the experience of nurses caring for patients with delirium. Future research should focus on identifying how the nurses cope with these challenges and build their confidence and knowledge of caring for patients with delirium. By identifying what has happened in the past, what strategies they have used will allow the development of concrete approaches to overcome current identified overlaps and gaps. In addition, there remains opportunities for research to be conducted to develop patient assessment tools, assessing the efficacy of the tools, best nurse education approaches in the use of these tools, and in what capacity these tools can assist in the decision-making of and care for people with delirium.

### 4.2. Implications for Practice

The findings from this review reveal the need for education among nurses regarding delirium and the best-practice care of these clients. Regular in-services and update education sessions will assist to provide nurses, in various clinical contexts or jurisdictions, with greater confidence in the assessment of delirium (i.e., Confusion Assessment Methods—CAM, Rapid Clinical Test for Delirium—4AT, etc.). In addition, such education will also increase nurses’ competence as they care for the acutely confused person. The development of new systems to identify the requirement of essential nursing services will enable appropriate skill mix and staffing levels to care for this population. Lastly, healthcare organisations may benefit from being further cognizant regarding the stress and anxiety encountered by nurses, when caring for patients experiencing delirium. Such recognition and systems will assist to inform how best to provide or put in place appropriate support strategies for nursing staff. These supports may include developing specific delirium nurse education that encompasses theory and clinical skills. In addition, improved processes, practices, and feedback systems will enable the reporting of delirium, near misses, and meeting care needs or addressing care issues. Lastly, the implementation of both formal and informal measures and system in place that enable greater teamwork, shown to improve patient and nursing outcomes, when caring for those with delirium [12,19,25,32,33].

### 4.3. Limitations of the Review

Given the systematic review examined qualitative research English speaking Western countries, the findings may not be representative of nursing patients with delirium globally. Although insightful, additional emphasis may benefit from the inclusion of quantitative research which may be addressed as part of future reviews. Lastly, the lack of guidance from an experienced biomedical information specialist may have implications regarding the search strategy undertaken and achieved.

## 5. Conclusions

Delirium is a condition that must be identified at the earliest possible moment to avoid complications and long-term cognitive dysfunctions. Nurses must possess both requisite knowledge and skills, to care for patients with delirium. However, in the absence of critical knowledge or specifically honed skills in the care of the confused patient, key strategies such as working with and being supported by peers and a patient’s family members are positive strategies, while restraint should be used a last resort. Such positive strategies can be easily put into place to facilitate appropriate and safe care until healthcare systems and systems approaches become more concrete and tangible. Overall, the review enlightens the need for increased delirium education, including coping strategies to effectively care for patients with delirium and improve clinical practice.

## Figures and Tables

**Table 1:** Characteristics of the studies.

Authors	Purpose	Participants	Methods	Analysis
Agar et al., [16]	To explore nurses’ assessment and management of delirium when caring for people with cancer, the elderly or older people requiring psychiatric care in the inpatient setting.	*n* = 40 registered nurses working in public hospital inpatient dedicated units in palliative care, aged care, aged care psychiatry or oncology in South West Sydney, Australia.	Open ended semi-structured interviews.	Thematic content analysis.
Hosie et al., [18]	To explore the experiences, views and practices of inpatient palliative care nurses in delirium recognition and assessment.	*n* = 30 nurses from nine specialist palliative care inpatient services, Australia	Critical incident technique and semi-structured interviews.	Thematic content analysis
Kjroven et al. [15]	To examine the language practices and discourses that shape and discipline nurses care for patients with post-operative delirium.	*n* = 6 nurses working in a surgical ward in Canora Jubilee Hospital, Canada.	Face to face in-depth interviews	Foucauldian post structural/postmodern model and Content analysis
Hosie et al., [18]	To identify was to identify nurses’ perceptions of the barriers and enablers to recognising and assessing delirium symptoms in palliative inpatient settings.	*n* = 31 nurses from 9 specialist palliative care inpatient services in Australia.	Semi structured questionnaire.	Thematic analysis.
Yue et al., [19]	To explore the experiences of nurses caring for patients with delirium in ICU in China.	*n* = 14 ICU nurses in Beijing, China.	Semi-structured	Thematic analysis.
Zamoscik et al., [20]	To explore nurses’ experiences and perceptions of delirium, managing delirious patients, and screening for delirium, five years after introduction of the Confusion Assessment Method for Intensive Care into standard practice.	*n* = 12 nurses from a medical- surgical intensive care unit at a large teaching hospital in the United Kingdom.	qualitative interviews.	Thematic analysis
Brooke & Manneh, [25]	To explore the lived experiences of caring for a patient during an acute episode of delirium by nurses working in cardiology, elderly care, renal, or respiratory specialities.	*n* = 23 nurses were recruited, including nurses from: cardiology (*n* = 6), elderly care (*n* = 5), renal (*n* = 6), and respiratory (*n* = 6), UK.	Focus group discussions	Thematic analysis
LeBlanc et al., [26]	To explore the lived experience of ICU nurses caring for patients with delirium	*n* = 8 Participants in this study were recruited from two ICUs in a university- affiliated, tertiary care academic health care centre in Canada	Semi-structured interview	Thematic analysis
Kristiansen et al., [12]	To investigate nurses’ experiences of caring for older (65+ years) patients afflicted by delirium in a neurological department.	*n* = 14 nurses from the neurology department in Denmark.	Interview	Thematic analysis
Schmitt et al. [27]	To investigate common delirium burdens from the perspectives of patients, family caregivers, and nurses.	*n* = 15 nurses from an urban teaching hospital in Boston, Massachusetts, US	Focus groups and interviews	Thematic analysis

**Table 2:** Study findings.

Category	Summary	Credibility Rating	Illustration
Concept ambiguity	Nurses were unaware about the process and course of delirium, causing difficulties in understanding the patients.	Unawareness regarding process and course of delirium (U) ^1^	“It is difficult for me to distinguish delirium from other neurological disorders as I haven’t learned how to tell the difference. For example, temporal lobe damage also results in the same kind of restless movement as delirium. Therefore, I can’t tell if the symptoms are the result of cerebral haemorrhage or delirium. I don’t know what definition of delirium is.” (Yue et al., [19] (p. 5)).
Lack of knowledge and education	The deficit in knowledge and education created lack of confidence in nurses to provide appropriate care.	Knowledge deficit (U)	“I just think as nurses we are not trained enough in dealing with delirium.” (Hosie et al., [18] (p. 823)).
Resources and staffing	The shortage in staff and lack of resources, left the vulnerable and unsupported.	Level of staffing and other resources (C) ^1^	“… so whilst one person might help the nursing staff with that confused patient, that nursing staff member still has to deal with everything around that patient like medications, treatment…” (Agar et al., [16] (p. 892)).
Workload	Nurses experienced increased workload, and frustration from the workload, when caring for patients with delirium.	Unyielding workload (U)	“It means putting other things aside and treating the immediate needs. I have to divert my attention to helping them with whatever is happening right now.” (LeBlanc et al., [26] (p. 95))
Time restraints	The care of patients with delirium was time consuming and nurses felt they were unfair to the other patients	Lack of time (C)	“Some participants noticed that nurses often fail to undertake the test due to time constraints and that the results are not always reported to the doctors.” (Zamoscik et al., [20] (p. 96)).
			“It is sometimes extremely time consuming guiding them 100 times back to bed, and at the same time, I think that I have five other bells also ringing, and I actually need to go complete rounds on all my patients.” (Kristiansen, Konradsen & Beck, [12] (p. 924))
Stress and anxiety	Caring for patients with delirium generated stress, anxiety and mental conflicts in the nurses caring for them.	Nurses feeling pressured (C)	“Despite nurse doing her best to prevent patient from removing tubes, the incidence still leaves the nurse feeling very nervous. The nurses are always under pressure.” (Yue et al., [19] (p. 5)).
Nurse’s safety	Nurses were concerned and feared for their own safety.	Feeling unsafe (U)	“We had a lovely lady who became confused with a UTI, she was a completely different person, and she was verbally aggressive, she did try to throw things, pinch and punch, but we understood that she was confused” (Brooke et al., [25] (p. 5))
Patient’s safety	Patient safety was a prime priority for all nurses.	Ensuring patient safety (U)	“I’m always concerned about their safety when I go in and they are confused, not directable.” (Kjorven et al., [15] (p. 330)).
Constant surveillance	Staying with the patient constantly to ensure safety of the patient.	Closely monitoring and following the patient (C)	“Specials (one on one nursing) was thought an ideal strategy…” (Agar et al., [16] (p. 892)).
Restraints	The use of physical and chemical restraints to control the confused patient.	Use of side rails and sedatives (C)	“Non-pharmacological interventions were highly valued…” “Bed rails were sometimes helpful” (Agar et al., [16] (p. 892)).
Family support	Family members play a vital role in the management of delirium and provide support to the nurses caring for patients with delirium.	Role of family members in calming the patient (C)	“Sometimes, we call the family member and ask them to come to the ICU to comfort the patient. This approach works well. As soon as the patients see their family members, they calm down and regain their consciousness.” (Yue et al., [19] (p. 6)).
Support from peers	Nurses are supported by their peers in care of patients with delirium and learn from the experience of other nurses.	Peer nurses were involved in decision making (U)	‘‘We sat down and we talked about the behaviours that had been happening over the last few days.” (Hosie et al. [28] (p. 1360))

^1^ U = Unequivocal; C = Credible.
